# A Parallel Nonrigid Registration Algorithm Based on B-Spline for Medical Images

**DOI:** 10.1155/2016/7419307

**Published:** 2016-12-07

**Authors:** Xiaogang Du, Jianwu Dang, Yangping Wang, Song Wang, Tao Lei

**Affiliations:** ^1^School of Electronic & Information Engineering, Lanzhou Jiaotong University, Lanzhou 730070, China; ^2^Lanzhou Yuxin Information Technology Limited Liability Company, Lanzhou 730000, China; ^3^College of Electrical & Information Engineering, Shaanxi University of Science & Technology, Xi'an 710021, China

## Abstract

The nonrigid registration algorithm based on B-spline Free-Form Deformation (FFD) plays a key role and is widely applied in medical image processing due to the good flexibility and robustness. However, it requires a tremendous amount of computing time to obtain more accurate registration results especially for a large amount of medical image data. To address the issue, a parallel nonrigid registration algorithm based on B-spline is proposed in this paper. First, the Logarithm Squared Difference (LSD) is considered as the similarity metric in the B-spline registration algorithm to improve registration precision. After that, we create a parallel computing strategy and lookup tables (LUTs) to reduce the complexity of the B-spline registration algorithm. As a result, the computing time of three time-consuming steps including B-splines interpolation, LSD computation, and the analytic gradient computation of LSD, is efficiently reduced, for the B-spline registration algorithm employs the Nonlinear Conjugate Gradient (NCG) optimization method. Experimental results of registration quality and execution efficiency on the large amount of medical images show that our algorithm achieves a better registration accuracy in terms of the differences between the best deformation fields and ground truth and a speedup of 17 times over the single-threaded CPU implementation due to the powerful parallel computing ability of Graphics Processing Unit (GPU).

## 1. Introduction

Image registration is a process used in medical image analysis to determine a spatial transformation that aligns image data according to the spatial coordinate of pixels. The process involves a reference image and a moving image; the moving image is deformed by a spatial transformation to align with the reference image [1]. With the development of medical imaging technologies, it is easy to obtain many images with different modalities and gain a more complete understanding of anatomy and function by registering and fusing these images. Generally, according to spatial transformation, image registration is categorized into two types: rigid registration and nonrigid registration. The rigid registration only describes the motion which is limited to global rotations and translations, and the nonrigid registration always includes very complex local and elastic deformations except rigid transformation.

Practically, nonrigid registration is always widely applied in the many clinical procedures to improve the geometric precision, such as disease diagnostic [2], neurosurgery [3] and Image-Guided Radiation Therapy (IGRT) [4]. Up to present, several classical nonrigid registration algorithms have been proposed and improved in the performance of registration results, including viscous fluid registration [5], demons registration [6], Finite Element Model (FEM) [7] and B-spline registration [8]. B-spline registration always employs uniform cubic B-spline curves to determine a displacement vector field which maps voxels in the moving images to those in the reference images [8]. Each voxel's displacement between the moving image and the reference image is parameterized in terms of uniformly spaced control points that are aligned with the voxel mesh, and displacement vectors are obtained via interpolation of the control-point coefficients using piecewise continuous B-spline basis functions. Recently, B-spline registration became very popular because of their flexibility and robustness and is therefore able to perform both monomodality and multimodality image registration. However, In the B-spline registration algorithm, there are three time-consuming steps: B-spline interpolation, similarity metric computation, and similarity metric gradient computation. First, in the process of B-spline interpolation, a coarse array of B-spline coefficients is considered as the input and a fine array of displacement values is computed as the output defining the deformation vector field from the moving image to the reference image. Therefore, B-spline interpolation is expensive in terms of executing time for the fine deformation mesh with many control points. In addition, similarity metric is a very crucial step to measure the registration results, and it has an important influence on the accuracy of the registration results. In addition, similarity metric is calculated in each iteration of the B-spline registration algorithm, and its computational efficiency has a serious impact on the performance of the whole registration process. Finally, similarity metric gradient computation requires evaluating the partial derivatives of the similarity metric with respect to each spline-coefficient value of all control points; it is therefore a very time-consuming step to obtain better accuracy of registration results in the case of much more control points. In summary, the B-spline registration algorithm always needs more execution time for the larger amount of medical image data, which limits the clinical applications of B-spline registration [9, 10].

Aiming to improve the accuracy and significantly accelerate B-spline registration algorithm, we develop a B-spline-based nonrigid registration algorithm that is suitable for executing on GPU by considering the LSD as similarity metric. In our algorithm, all of the computation-intensive tasks are designed and implemented on GPU using the parallel computing framework, Computing Unified Device Architecture (CUDA). Experiments show that our algorithm improves the accuracy of registration results and achieves a speedup ratio of 17 times comparing to the single-threaded serial implementation. The main contributions are summarized as follows:

1. We introduce the LSD as similarity metric in the B-spline registration algorithm to obtain the higher registration accuracy for medical images.
2. We design a parallel computing strategy and create LUTs for B-splines registration to improve the execution performance. Meanwhile, three time-consuming steps including B-splines interpolation, the similarity metric LSD, and its gradient computation are designed in the form of the kernel functions which are executed in parallel on GPU.

The rest of this paper is organized as follows.  Section 2 presents the related works of accelerating B-spline registration using cluster, many-core and GPU.  Section 3 describes our B-spline registration algorithm employed the LSD as the similarity metric and NCG optimization.  Section 4 explains the parallel computing details of our proposed algorithm based on CUDA.  Section 5 shows experiments results and analysis. Finally, Section 6 concludes our work.

## 2. Related Work

The parallel computing technology is an effective way to accelerate many general purpose applications and has a very crucial influence on the field of high performance computing. Parallel operations can be performed independently on different partition of medical images due to the good data parallel characteristic of medical images. At present, parallel computing has been widely used in medical image processing [11, 12], for example, image registration [13, 14]. There are three parallel computing technologies for accelerating medical image registration: cluster, many-core, and GPU computing.

Cluster computing is a well-established technology of accelerating image processing algorithms and nonrigid registration is also no exception. The main strategy of cluster computing is composed of two stages: dividing the medical image into many different small partitions and processing them independently on different computers while minimizing the communication cost. Zheng et al. [15] used mutual information as a similarity metric and developed parallel nonrigid registration algorithms on a computer cluster. Results reported in Zheng et al. [15] for B-splines show a speedup of *n*/2 for *n* processors compared to a sequential implementation.

The recent developments in many-core processor technology offer new opportunities for truly large-scale and cost-effective parallel computing on a single chip. Meanwhile, multiprocessor computing techniques are utilized for accelerating image registration algorithms. Jiang et al. [16] used a FPGA-based implementation leading to a speedup of 3.2 times compared with a 2.666 GHz CPU execution. Rohlfing and Maurer Jr. [17] reduced computation time more than 50 times by using 64 CPUs of a shared memory supercomputer. More recently, Rohrer et al. [18] presented a multicore implementation of the B-spline computation based on a Cell Broadband Engine platform, reporting a speedup of 40 times over a sequential CPU implementation. Although these techniques provide considerable computation time improvements, they require either high technical knowledge or hardware with prices inhibiting wide adoption.

Modern GPU is the extension of many-core technology and has a large number of cores on the chip, and this many-core architecture of GPU is more suitable for parallel computing. For algorithms that can be parallelized within the SIMD model, a single GPU offers the computing power equivalent to a small cluster of desktop computers. As noted in the introduction, a bottleneck of the B-spline FFD registration algorithm is the cubic B-spline computation. Consequently, some work has been done to speed up this part by using GPU. For example, Sigg and Hadwiger [19] developed a cubic B-spline interpolation method utilizing linear interpolation of the GPU hardware; however, such an approach suffers from the limitations imposed by the low fixed-point precision of the hardware interpolator as reported by Ruijters et al. [20], which limits its applicability to robust and precise medical image registration.

In addition, researchers proposed many improved approaches to accelerate B-spline registration algorithm with respect to B-spline interpolation and similarity metric computation. Modat et al. [21] presented a parallel-friendly formulation algorithm that is suitable for GPU, it only take less than 1 min to perform registration of T1-weighted MR images without reducing the registration accuracy. Shackleford et al. [22] employed the SSD as similarity metric and accelerated B-spline registration within the stream-processing model using GPU, and this algorithm achieved a good speedup over the single-threaded CPU. Ikeda et al. [23] proposed an efficient GPU-based acceleration method for the nonrigid registration of multimodal images, and their main contribution is efficient utilization of on-chip memory for Normalized Mutual Information (NMI). Ellingwood et al. [24] adopted the sum of squared tissue volume difference as a similarity metric and thus developed a multilevel B-spline transform method to implement nonrigid mass-preserving registration of two CT lung images on GPU, and this method outperforms its multithreaded CPU version in terms of running time.

## 3. Our Algorithm

Aiming to significantly improve the accuracy and the performance of B-spline registration algorithm, we propose a CUDA-based parallel B-spline registration algorithm which employs LSD as the similarity metric and used NCG as optimization strategy. In this section, we describe the details of our B-spline nonrigid image registration algorithm. Firstly, we represent the theory of describing nonrigid deformation using the cubic B-spline basis function. Secondly, we introduce the LSD as the similarity metric of our algorithm. Finally, we explain the NCG optimization procedure and the gradient of similarity metric in our algorithm.

### 3.1. B-Spline Free-Form Deformation

B-spline FFD is used as the nonrigid transformation model, and three-dimensional (3D) deformation field is defined by 3D mesh of control points in the Cartesian coordinates. The deformation field for a voxel located at coordinates (*x*, *y*, *z*) in the reference image can be mathematically described as follows [15, 21]:

(1) $$\begin{array}{c} \overset{\to }{T}\left(\;x,y,z\;\right)=\overset{3}{\underset{i=0}{\sum }}\;\overset{3}{\underset{j=0}{\sum }}\;\overset{3}{\underset{k=0}{\sum }}B_{i}\left(\;u\;\right)B_{j}\left(\;v\;\right)B_{k}\left(\;w\;\right)\phi \left(\;l,m,n\;\right). \end{array}$$

In (1), *B*_*i*_(·) is the cubic B-spline basis function in the *x* direction [8], and *ϕ* represents the deformation mesh. In the 3D medical image data, a partition region is composed by the eight adjacent control points, and the 3D spacing coordinate of this partition region is shown as follows:

(2) Ωx=xNx−1,Ωy=yNy−1,Ωz=zNz−1,

where *N*_*x*_, *N*_*y*_, and *N*_*z*_ are the voxel numbers of the *x*, *y*, and *z* directions of the control point mesh. Displacement of voxels in each region *Ω*(*x*, *y*, *z*) are affected around 64 control points. The 64 control points coordinates CP(*l*, *m*, *n*) are calculated in the following scheme [8, 25]:

(3) CPl=xNx−1+i,CPm=yNy−1+j,CPn=zNz−1+k.

In the medical image volume data, the voxel with the coordinate (*x*, *y*, *z*) has the local coordinate Local(*u*, *v*, *w*) in the region *Ω*(*x*, *y*, *z*), and the value is normalized in [0,1] as follows [8, 25]:

(4) Localu=xNx−xNx,Localv=yNy−yNy,Localw=zNz−zNz.

### 3.2. The Similarity Metric LSD

A similarity metric quantifies the degree of alignment and is used to optimize the transformation's parameters to achieve maximal alignment. The similarity metric has a key influence on the accuracy of registration results. Sum of Squared Difference (SSD) is a very classical similarity metric based on pixel computation, and it is widely used in the monomodality medical image registration. However, when SSD is used as the similarity metric, due to the fact that the SSD values of the different iterations are very closed in the later stage of optimization process, it leads to appear premature convergence rate and also affects the accuracy of the registration results.

Since the function value of the logarithm transformation has different influence on the different intervals of the definition domain, especially when the independent variable value is more and more small, the function value changes more quickly. In other words, the difference between two values of logarithmic function is more sensitive to the interval which has a small logarithmic values than the interval which has a large logarithmic values. In the latter stage of the registration process, the difference between the reference image and the moving image subject to local deformation becomes much smaller, the logarithmic operation can make the SSD of similarity metric to change more quickly, and it is helpful to find best deformation mesh more effectively. Therefore, in order to further improve the accuracy of registration results, we perform logarithmic operation on SSD and introduce the LSD as the similarity metric of the B-spline registration algorithm. LSD can be mathematically described as follows:

(5) LSD=1NxNyNz·∑x=1Nx ∑y=1Ny ∑z=1Nzln⁡Fx+Tx,y+Ty,z+Tz−Rx,y,z2+1.

In (5), *R*(*x*, *y*, *z*) represents the reference image, *F*(*x* + *T*_*x*_, *y* + *T*_*y*_, *z* + *T*_*z*_) is the moving image with B-spline deformation, and *N*_*x*_, *N*_*y*_, and *N*_*z*_ are separately the voxel numbers in the *x*, *y*, and *z* directions of reference and moving images. In order to promote smooth deformation, a penalty term *C*_smooth_ is added to the LSD value. The final cost function *C* to be optimized is a balance between the LSD and the deformation penalty:

(6) $$\begin{array}{c} C=\left(\;1-\lambda \;\right)\times LSD-\lambda \times C_{smooth}, \end{array}$$

where 0 ≤ *λ* < 1. The penalty-term *C*_smooth_ was first used for image registration by Rueckert et al. [8].

### 3.3. B-Spline Registration Optimization Based on NCG

Conjugate Gradient (CG) is a very effective optimization method for optimization problem. CG overcomes the shortcoming of slow convergence of the gradient descent method and also avoids the shortage from Newton's method, which needs to store and compute the Hessian matrix and the inverse matrix [26]. The NCG method is an extension of CG and suitable for minimizing general nonlinear functions. Due to the fact that the B-spline registration process is a nonlinear optimization problem, the NCG method is adopted for the entire registration process optimization in our algorithm. So it is important to calculate the gradient of the similarity metric for control points in every iteration and update the control point mesh according to the negative gradient direction from the previous search. Iteratively, the process of updating parameters of control point mesh can be mathematically described in

(7) $$\begin{array}{c} \phi_{i+1}=\phi_{i}-\alpha_{i}d_{i}\;i=1,2,3,\ldots , \end{array}$$

where *ϕ*_*i*_ = (*ϕ*_*x*_, *ϕ*_*y*_, *ϕ*_*z*_) denotes the displacement vector of control points in the iteration process. *i* is the iteration number. *α*_*i*_ is a scalar, which represents the displacement magnitude of the negative gradient direction, and is obtained using the inexact linear search method. *d*_*i*_ represents the search direction, which is calculated as

(8) $$\begin{array}{c} d_{i}=\left\{\begin{array}{cc} -\frac{\partial C}{\partial \phi_{i}} & \text{for }i=1\\ -\frac{\partial C}{\partial \phi_{i}}+\beta_{i}d_{i-1} & \text{for }i\geq 2, \end{array}\right. \end{array}$$

where *β*_*i*_ is a variable, which is calculated as

(9) βi=max⁡0,min⁡βiHS,βiDY,

(10) βiHS=∂C/∂ϕiT×∂C/∂ϕidi−1∂C/∂ϕi−∂C/∂ϕi−1T

As seen in (10), we thus require the derivative ∂*C*/∂*ϕ* of the cost function:

(11) $$\begin{array}{c} \frac{\partial C}{\partial \phi }=\left(\;1-\lambda \;\right)\times \frac{\partial LSD}{\partial \phi }-\lambda \times \frac{\partial C_{smooth}}{\partial \phi }. \end{array}$$

In order to decrease the computational complexity, we propose a voxel-centric approach to evaluate the point-centric gradient using the chain rule. We first compute the gradient for every voxel and then gather the information from all voxels to compute the gradient for control points. In (11), ∂LSD/∂*ϕ* is calculated via the chain rule as

(12) $$\begin{array}{c} \frac{\partial LSD}{\partial \phi }=\frac{1}{N_{x}N_{y}N_{z}}\overset{N_{x}}{\underset{x=1}{\sum }}\;\overset{N_{y}}{\underset{y=1}{\sum }}\;\overset{N_{z}}{\underset{z=1}{\sum }}\frac{\partial LSD}{\partial \overset{\to }{T}}\times \frac{\partial \overset{\to }{T}}{\partial \phi }, \end{array}$$

where $\partial LSD/\partial \overset{\to }{T}$ denotes the partial derivative of similarity metric for voxels, and it is calculated as follows:

(13) ∂LSD∂T→x,y,z=2×∇Fx,y,z×Fx+Δx,y+Δy,z+Δz−Rx,y,z1+Fx+Δx,y+Δy,z+Δz−Rx,y,z2,

where *F*(*x* + Δ*x*, *y* + Δ*y*, *z* + Δ*z*) is the moving image after the deformation, *R*(*x*, *y*, *z*) is the reference image, and ∇*F*(*x*, *y*, *z*) denotes the initial gradient of the moving image. In (12), the second term is the derivative value of deformation field with respect to a control point, and the calculation form is described in

(14) $$\begin{array}{c} \frac{\partial \overset{\to }{T}}{\partial \phi }=\overset{3}{\underset{l=0}{\sum }}\;\overset{3}{\underset{m=0}{\sum }}\;\overset{3}{\underset{n=0}{\sum }}B_{l}\left(\;u\;\right)B_{m}\left(\;v\;\right)B_{n}\left(\;w\;\right). \end{array}$$

In (14), $\partial \overset{\to }{T}/\partial \phi$ is the only metric related to the B-spline basis function, and it is also a constant value when the local coordinates of voxels in the mesh partition are determined. In order to simplify the parallel computing process of our algorithm, we set *λ* to 0.

## 4. Parallel Accelerating Procedure for Our Algorithm

To effectively improve the execution efficiency of the B-spline registration, we employ the GPU to accelerate the time-consuming steps of our algorithm. In this section, we present the parallel computing details of our algorithm using the GPU. First, we explain the main procedure of our algorithm based on GPU. Secondly, we develop the parallel computing strategy of B-spline registration based on the characteristic of locality of B-spline transformation and design the LUTs for storing the reusable data for parallel kernel functions executing on the GPU. Then, we present kernel functions for these time-consuming steps. Finally, we summarize our proposed parallel algorithm based on GPU.

### 4.1. The Main Procedure of Our Algorithm

In general, data parallelism is the main requirement for an algorithm to benefit from GPU execution. Our nonrigid registration algorithm based on B-spline FFD comprises three components, which may be considered independently: transformation of the moving image using the splines interpolation, evaluation of a similarity metric, and optimization against this similarity metric. In these three components, the optimization process always requires adequate iterations and the current iteration is always dependent to the previous iteration, so it is serial process and is not implemented in parallel on the GPU. Individually, B-splines interpolation and evaluation of a similarity metric may be formulated in a data parallel manner as they mainly consist of voxel-wise computations. In our algorithm, NCG is used for the optimization, so the first order derivative of the similarity metric is computed to determine the optimal direction of the next iteration. Since the LSD is used as the similarity metric for evaluating the registration quality in our algorithm, we also design the parallel kernel function of the gradient of similarity metric LSD in terms of the highly parallel data features. The main framework of our algorithm using GPU is shown in Algorithm 1.

### 4.2. Parallel Computing Strategy for B-Spline Registration

**Figure 1:**
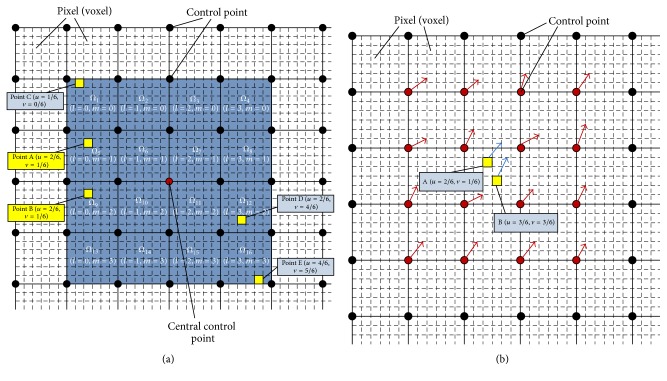
The deformation of B-spline mesh deformation in 2D image data. (a) shows the displacement vector calculation of central control point. (b) illustrates the displacement vector calculation of pixels in a local region. The black points are control points, and the deformation mesh is constituted by all the black control points; the dashed square is a pixel and the image data is constituted by all the dashed squares; the displacement values in the *X* and *Y* directions can be optimized for each control point. The spacing of control point mesh includes six pixels in the *X* and *Y* directions.

In the B-spline registration algorithm, the elastic deformation of the moving image is uniform cubic B-spline transformation which is parameterized by control point mesh.  Figure 1 shows that the elastic deformation of the 2D moving image is described using control point mesh. In Figure 1(a), since the red control point is the current central control point of 4 × 4 regions, the displacement vector of the central control point will be established by the around 4 × 4 regions and is entirely unrelated to the other regions of the moving image; in Figure 1(b), the displacement vector of each pixel located in the partition region is determined by its neighboring 4 × 4 control points when updating the B-spline transformation on the moving images. In other words, relative displacement changes of any control points only affect the pixels in the local region and are independent of the other global pixels of the moving images, so B-spline transformation has a good ability to describe the local elastic deformation of moving images. Therefore, this locality characteristic of B-spline transformation is a key foundation for designing the parallel computing strategy.

To improve effectively the executing speed of our B-spline registration algorithm using GPU, we develop a parallel computing strategy for B-spline registration and create LUTs to store the reusable data in the parallel processing procedure. Our parallel computing strategy is dividing the moving images into many different small and nonoverlapping partition regions in terms of the locality characteristic of B-spline transformation and then designing the parallel kernel functions to process them independently by many different threads on the different cores of GPU while utilizing the different memory of GPU effectively and minimizing the communication cost of thread blocks.

In order to ensure that the registration process on the GPU can effectively be executed in parallel, the following initialization needs to be done on the CPU before the entire process: (1) image volume data is loaded into host memory and copied from the host memory to the GPU memory; then bounding it to the texture memory of the GPU can improve the data access efficiency; (2) the initial B-spline mesh parameters are loaded from the host memory into the global memory of the GPU; (3) we precompute some reusable values, such as the B-spline basis function products, which are constructed and stored as LUTs in the texture memory of the GPU. In terms of the symmetry of volume data, we can create three LUTs for the parallel process as follows:

(1) The lookup table LUT_bfp_ of basis function products. For the same normalized local coordinate (*u*, *v*, *w*), we precompute basis function products *B*(*u*) × *B*(*v*) × *B*(*w*) and store them in the LUT_bfp_, which can be reused in the kernel functions of computing similarity metric and the gradient of similarity metric. Therefore, it can avoid repeated calculations and effectively improve the computational speed. For example, in Figure 1(a), the two pixels (A and B) are in their respective local partition regions with the same local coordinate, so they can reuse the same value of B-spline basis function products.
(2) The lookup table LUT_cpi_ of control point indexes. Since all voxels in the same region compute their displacement vectors using the same 64 control points, we precompute the index set of control points in the same region and create this LUT for storing them to improve the executing efficiency.
(3) The spatial gradient look-up table LUT_gradient_ of moving images. Because the gradient of the initial moving image remains the same value in the entire registration process, we precompute the gradient of moving images on the CPU and create a LUT to save them in the texture memory of GPU, which can reduce the complexity of parallel calculation.

### 4.3. Parallel Kernel Functions of Our Algorithm

In every iteration of our algorithm, B-spline transformation, similarity metric, and the gradient of similarity metric are three time-consuming steps. In terms of the above parallel computing strategy, we need to design three kernel functions for these time-consuming steps, and these kernel functions are executed in parallel on the GPU by multiple threads to improve the execution efficiency of our B-spline image registration algorithm. Since B-spline transformation and similarity metric computation are calculated for each voxel of the moving images, we only design a parallel kernel function which sequentially calculates these two steps.

#### 4.3.1. B-Spline Transformation and Similarity Metric Computation

We use the LSD as the similarity metric to evaluate the quality of deformation field in the proposed algorithm. As described in Section 3.2, LSD accumulates the square of the intensity difference between the reference images and the moving images subject to the deformation field. In consideration of the computation of LSD with a good parallelization, we design a kernel function* kernelFunctionSimilarity()* to compute LSD and B-spline transformation in parallel on the GPU in this paper. The kernel function is illustrated in Algorithm 2.

#### 4.3.2. Similarity Metric Gradient for Control Points

As shown in Figure 1, the displacement of each voxel in the same region is affected by displacements of 16 control points around the region when the moving image is subject to B-spline deformation. We compute the partial derivatives of all voxels for each control point in the same region, and all voxels housing in the same region have the contributions to 16 control points around this partition region, so each thread is corresponding to a partition region rather than a control point to improve the executing performance of the kernel function on the GPU. The kernel function* CalSimilarityGradientKernelFunc()* calculating the gradient of similarity metric for control points is designed as Algorithm 3.

### 4.4. Summary of the Proposed Algorithm

**Figure 2:**
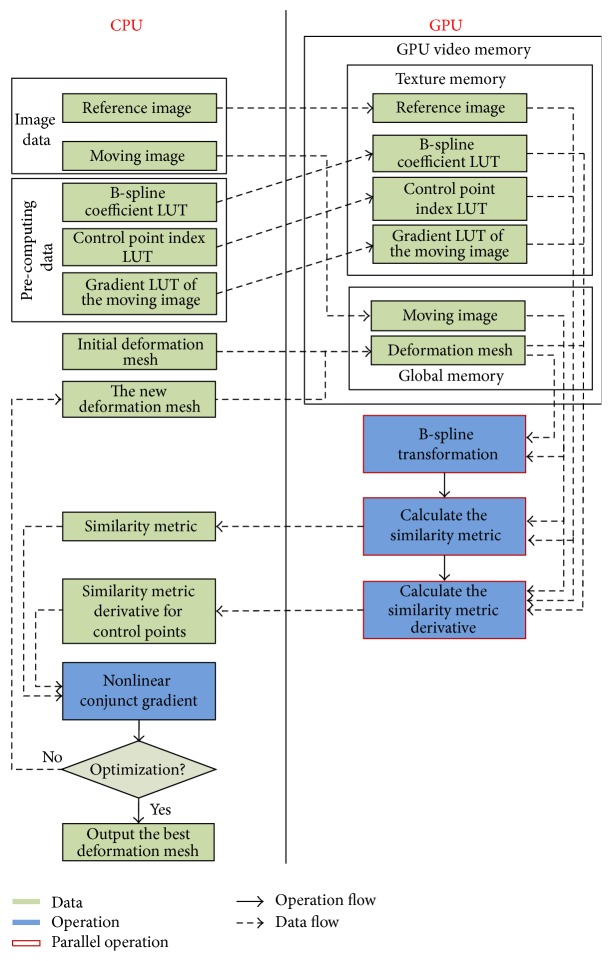
The parallelization process of the proposed algorithm. Light green areas represent the data required in the registration process, blue areas describe major steps of our algorithm, dashed arrows indicate the directions of data flow, and the solid arrows illustrate the directions of calculation processes.

Our algorithm is summarized and illustrated in Figure 2. In the beginning of the registration process, the image data, precomputing LUTs, and the initial B-spline mesh deformation parameters are loaded from the CPU to the GPU. More specifically, in order to improve the efficiency of accessing the memory in GPU, reference images and precomputing LUTs are bound to the texture memory of the GPU, and the initial B-spline mesh data and moving images are loaded into the global memory of the GPU; secondly, two kernel functions are executed in parallel by multithreading on the GPU, which are designed to calculate the B-spline transformation for the moving images according to the deformation mesh, the similarity metric, and the similarity metric gradient for control points respectively; and finally the similarity metric and its gradient values are transferred from the GPU to the CPU for the NCG optimization; the optimal B-spline deformation mesh is outputted when the optimized condition was meted; otherwise update B-spline deformation mesh parameters and continue to repeat the above registration process until the optimal values are found.

## 5. Experiment Results and Discussion

We evaluated the performance of the proposed algorithm on a medical imaging dataset that was obtained from the database of retrospective image registration evaluation (RIRE) at the Vanderbilt university [27]. This dataset contains a lot of medical image volume data of different patients from several protocols, including CT, T1-weighted (MR-T1), T2-weighted (MR-T2), and proton density (MR-PD) with a variety of slice thickness. All the corresponding slices from different protocols are originally aligned with each other. We implemented the proposed algorithm using CUDA C in the Microsoft Visual Studio  .NET 2010 and used CUDA6.0 of driver and runtime libraries [28]. The experimental hardware configuration is shown in Table 1.

### 5.1. Accuracy Comparison with Different Similarity Metrics

**Figure 3:**
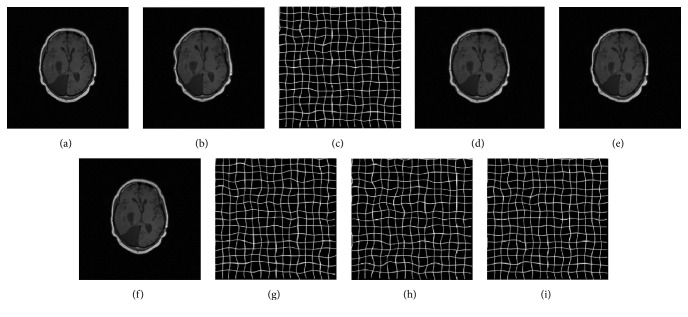
Accuracy comparison with different similarity metrics: (a) reference image; (b) moving image, which is distorted reference image with geometric distortion; (c) ground truth deformation field; (d)–(f) registered images using SAD-based, SSD-based and our algorithm, respectively; (g)–(i) estimated transformation using SAD-based, SSD-based, and our algorithm, respectively.

In the first experiment, to validate the registration accuracy of the proposed algorithm, we distorted the reference image MR-T1 with a known nonrigid geometric transformation field, or the so-called ground truth. Then, we applied the proposed algorithm and compared the registration results to the other two B-spline registration algorithms using Sum of Absolute Difference (SAD) and SSD as a similarity metric, respectively, which are implemented in the Insight Segmentation and Registration Toolkit (ITK) [29]. And finally, we compared the obtained deformations fields with the ground truth.  Figure 3 shows the registration results of three algorithms for brain MR-T1 images. Note that the registered moving images obtained by the proposed algorithm is visually more similar in deformation to the reference image than the images produced by the SSD-based algorithm and the SAD-based algorithm, respectively. Moreover, the estimated transformation field resulting from our algorithm is more similar to the ground truth than those obtained using the other two algorithms.

To measure the registration accuracy of the proposed algorithm, we computed the mean and standard deviation of the MSE between the ground truth and estimated displacement vectors.  Table 2 displays the MSE statistics of the estimated deformation, when compared to the ground truth. The first column shows the mean ground truth deformation, which represents the magnitude of the displacement vector that is used to generate moving images in each experiment. For each mean value of ground truth, eight different transformation fields with this mean are generated and applied to the reference image to generate the corresponding moving image. The other columns display the average and standard deviation of MSE obtained by using three different similarity metrics for the generated eight pairs of reference-moving images. The results obtained using the proposed algorithm are considerably small compared to those of SAD-based and SSD-based algorithm, and our algorithm shows the superior accuracy in comparison to the other two algorithms.

### 5.2. Accuracy Comparison with CPU Implementation

**Figure 4:**
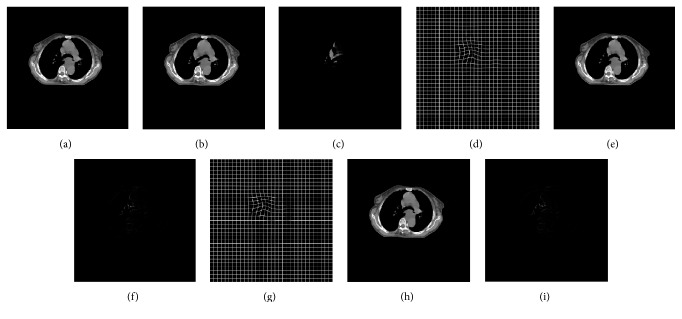
The registration results for chest images using the CPU-based and GPU-based implementation, respectively. (a) The reference image; (b) the moving image; (c) the difference result of the two images before registration; (d)–(f) are, respectively, the best deformation mesh, the moving image subjected to the best deformation, and the differential result after registration using CPU-based implementation; (g)–(i) are the best deformation mesh, the moving image subjected to the best deformation, and the differential result using our GPU-based implementation.

**Figure 5:**
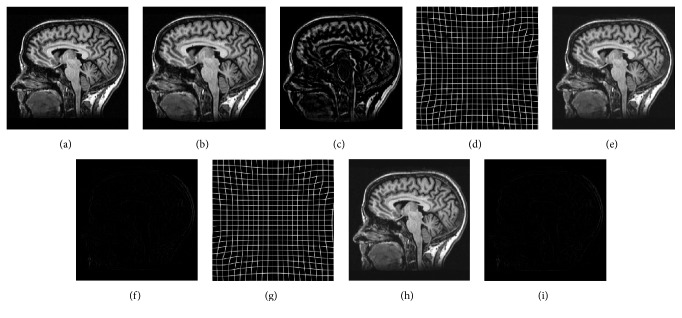
The registration results for brain images using the CPU-based and GPU-based implementation, respectively. (a) The reference image; (b) the moving image; (c) the difference between the reference and moving image before registration; (d)–(f) are, respectively, the best deformation, the moving image transformed with best deformation, and the difference image after registration using the CPU-based implementation; (g)–(i) are, respectively, the best deformation, the transformed moving image, and the difference after registration using our GPU-based implementation.

For our B-spline registration algorithm employing LSD as a similarity metric and NCG as a optimization strategy, we, respectively, implemented it based on CPU and GPU and compared the registration accuracy impact from the parallel computing framework for the four groups of medical images. Since the resolution of these images is 512 × 512, the control point mesh size, the number of control points, and the number of regions are 16 × 16, 35 × 35, and 32 × 32, respectively. In the GPU-based implementation of our algorithm, we set the grid size to 32 × 32 and the block size to 16 × 16 for the kernel function* kernelFunctionSimilarity()* and set the grid size to 4 × 4 and the block size to 8 × 8 for the kernel function* CalSimilarityGradientKernelFunc()*. Owing NCG is, respectively, adopted in the two implementations; we also set the maximum iteration number to 100 in the optimization process.  Figure 4 shows the results using two implementations for the first group of chest CT images.  Figure 5 shows the results that a reference image and moving image of the human head are matched using two implementations. As can be seen from Figures 4 and 5, the CPU-based implementation and the GPU-based implementation of our algorithm can both work well in describing the elastic deformation between reference and moving images, and they can both obtain highly accurate results.

In order to evaluate the accuracy of registration results, some researchers used some mathematical metrics. Ellingwood et al. [24] used the normalized Root Mean Square Error (RMSE) to mathematically evaluate accuracy of the GPU resultant displacement field against the single-threaded CPU implementation. RMSE is calculated as follows:

(15) $$\begin{array}{c} RMSE=\sqrt{\frac{{\sum_{x\in \Omega }\left\|v_{CPU}\left(x\right)-v_{GPU}\left(x\right)\right\|}^{2}}{\sum_{x\in \Omega }{\left\|v_{CPU}\left(x\right)\right\|}^{2}}}, \end{array}$$

where *v* denotes the resultant displacement field associated with a voxel at the coordinate *x*. To measure the registration quality, we generate the deformation field by running the registration process for 100 iterations and then compare the results against two CPU-based implementations (single-core and dual-core). The average RMSE for the four groups of medical images is 0.027 ± 0.011 comparing our GPU-based implementation against the single-core CPU implementation, and the average RMSE value is 0.024 ± 0.008 comparing our GPU-based implementation against the dual-core CPU implementation, so our GPU-based implementation and two CPU-based implementations can achieve almost the same accuracy of image registration. To sum up, according to two aspects of the image registration results and the RMSE metric, the experimental results show that both the two CPU-based implementations and our GPU-based implementation can generate near-identical vector fields with respect to the reference images and almost achieve good registration results with the same precision.

### 5.3. Execution Speed Evaluation

In order to evaluate and compare the registration performance, five different algorithm versions are implemented: (1) the single-threaded CPU implementation; (2) the dual-core CPU implementation using OpenMP; (3) the basic GPU implementation (this GPU version has a straightforward and naive kernel function that calculates the gradient of similarity metric for each control point); (4) the GPU implementation without LUTs (this version designs kernel function that calculates the gradient of similarity metric for each partition rather than each control point to decrease the calculation complexity, but it computes temporarily some intermediate values such as basis function products and does not still use LUTs); (5) our algorithm (our algorithm computes three time-consuming steps on the GPU and uses three LUTs and instruction optimization strategy in kernel functions to maximize execution performance).

**Figure 6:**
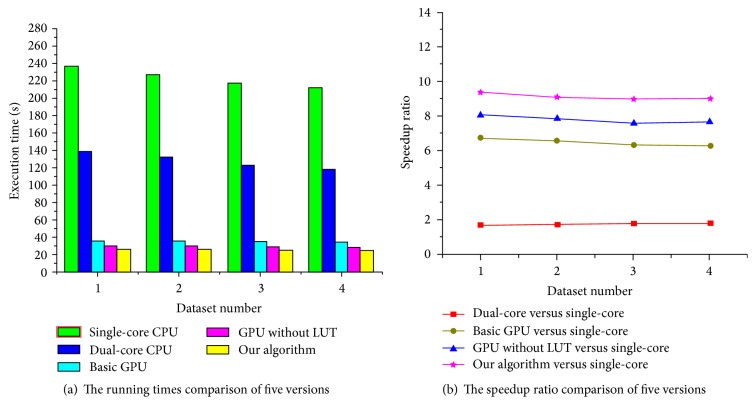
The performance comparisons of five algorithms for 2D images.

These versions are performed for four groups of medical images. In our algorithm, we first set the maximum iteration number to 100 and define the initial mesh spacing size to 10 × 10. Then, we set the grid and block size for the two kernel functions* kernelFunctionSimilarity()* (the grid and block size are 32 × 32 and 16 × 16, respectively) and* CalSimilarityGradientKernelFunc()* (the grid and block size are, respectively, 5 × 5 and 10 × 10). The performance of our algorithm is compared with the two CPU versions and two GPU versions under two measures: total execution times and speedup ratio. The speedup ratio is a persuasive measure used to compare the performance of GPU to CPU, which is mathematically described as speedup = time_CPU_/time_GPU_, where time refers to the total execution times. The execution times and speedup ratio comparisons of five versions for 2D images are, respectively, illustrated in Figure 6.

As shown in Figure 6, our algorithm requires approximately 25 s to complete the registration process for the reference and moving images with the size of 512 × 512. By comparing the execution time of our algorithm with the single-core CPU version (the total time is about 220 s) and the dual-core CPU version (the total time is about 130 s), the running speed of our algorithm is around 9 times faster than the single-core CPU version and 5 times faster than the dual-core CPU version. In addition, our algorithm is about 10 seconds less than the basic GPU version in terms of running time, and our algorithm is about 4 seconds less than the GPU version without LUTs. In Figure 6, the results show that our algorithm reduces the calculation complexity and accelerates the speed of image registration effectively due to employing the parallel computing strategy and LUTs.

**Figure 7:**
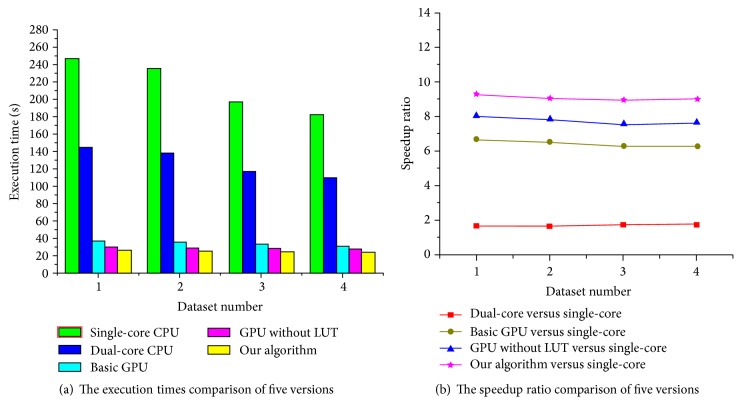
The performance comparisons of five algorithms for 2D images.

In the next experiment, we evaluate the impact of the initial B-spline mesh spacing size on the registration efficiency. For the reference and moving images with the size of 512 × 512, we first set the maximum iteration number to 100 and, respectively, set the initial B-spline mesh size to 8 × 8, 10 × 10, 16 × 16 and 32 × 32 for four groups of different images. Then, the grid and block size are, respectively, 32 × 32 and 16 × 16 for the kernel function* kernelFunctionSimilarity()* in our algorithm. In addition, B-spline spacing sizes in four various datasets require different numbers of control points and regions, so we set reasonable values for the grid and block sizes to adapt each of datasets for the kernel function* CalSimilarityGradientKernelFunc()*. Figure 7 illustrates the execution times and speedup ratio comparisons of five algorithm versions for different mesh spaces.

As shown in Figure 7, the running times of the single-core CPU version decrease about 60 s because control point mesh spacing size gets larger and the number of control points used to describe deformation field becomes less. The execution times of three GPU versions decreased less with the decrease of the amount of control points. Especially, in our algorithm, the mesh spacing size has a less impact on the execution speed of the entire registration process because the main time-consuming steps are executed by multithreading in parallel on the GPU.

**Figure 8:**
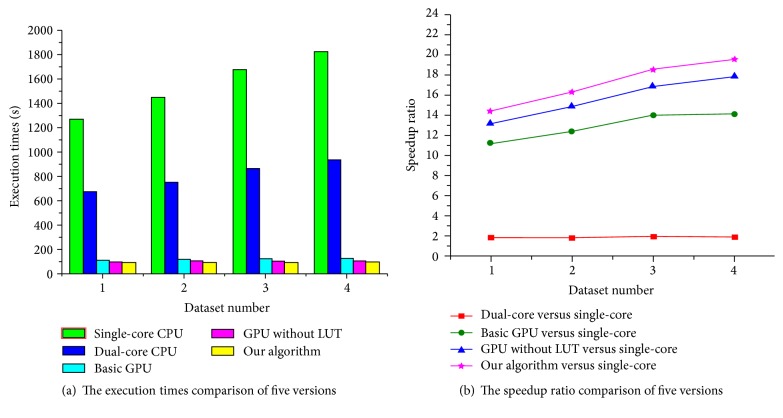
The performance comparisons of five algorithms for 3D images with different resolutions.

In order to evaluate the calculation efficiency of our algorithm for the different amounts of image volume data, we furthermore compare our algorithm with the other four versions in the elastic registration for four groups of 3D medical images with different sizes. The sizes of four group medical images are shown in Table 3. The execution time and speedup ratio comparison of five versions for 3D images with different resolutions are shown in Figure 8.

In Figure 8, experimental results show that the execution time of the single-core CPU version increases more significantly with the increasing amount of 3D volume data, but the execution time of our algorithm almost has no growth because of the powerful parallel computing ability of GPU. Meanwhile, our algorithm computes the gradient of the similarity metric for each partition rather than each control point to reduce the calculation complexity and improve the efficiency of global memory access, and we effectively create three LUTs and utilize kernel instruction optimization strategy in the design of kernel functions. Therefore, compared with the other two GPU versions, our algorithm utilizes the GPU parallel computing ability more fully, further improves the execution efficiency of B-spline registration, and especially achieves a better speedup ratio with the increasing amount of image volume data.

## 6. Conclusion

In this paper, we have introduced the LSD as similarity metric and developed a parallel computing strategy and some effective LUTs that greatly reduce the complexity of B-spline-based registration and then designed highly parallel kernel functions for computing the similarity metric and its gradient on GPU. We have demonstrated the accuracy and execution speed of our algorithm via experiments using the public medical image datasets. Experimental results highlight that our algorithm takes full advantage of the GPU parallel computing, and the entire B-spline nonrigid registration process achieved the speed ratio of 17 times for the large amount of volume data compared with the CPU-based implementation. The future work will focus on the parallel registration algorithm based on new similarity metrics for multimodality medical images.

## Figures and Tables

**Algorithm 1:**
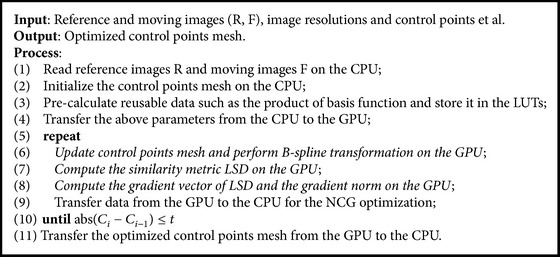
The GPU-accelerated B-spline registration based on LSD and NCG.

**Algorithm 2:**
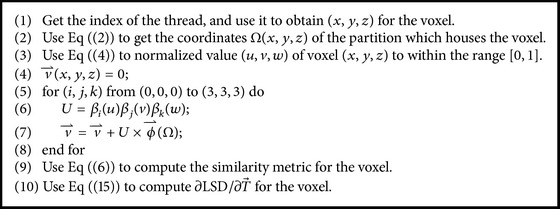
The pseudocode listing of the kernel function *kernelFunctionSimilarity()*.

**Algorithm 3:**
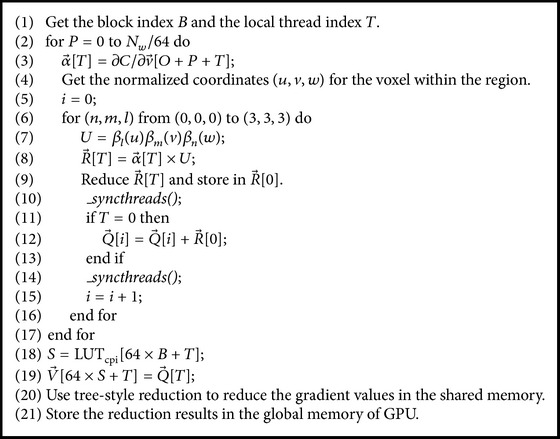
The pseudocode listing of the kernel function *CalSimilarityGradientKernelFunc()*.

**Table 1:** The experimental hardware configuration.

No.	Components	Parameters
1	CPU	Intel(R) Xeon(R) 1.90 GHz × 18
2	System memory	32 GB
3	Video card	Tesla K20m
4	Memory of video card	4 GB
5	CUDA cores	2496
6	GPU max clock rate	0.71 GHz
7	Memory clock rate	2600 MHz
8	Memory bus width	320-bit

**Table 2:** MSE statistics of the estimated deformation field.

Ground truth	MSE (average)			MSE (standard deviation)		
	SAD	SSD	Ours	SAD	SSD	Ours
2.0	0.652	0.547	0.521	0.057	0.036	0.034
3.2	0.765	0.576	0.563	0.062	0.051	0.046
4.4	0.932	0.789	0.766	0.065	0.044	0.026
5.3	1.391	0.915	0.887	0.078	0.037	0.031

**Table 3:** Four groups of 3D medical images with different sizes.

	Case 1	Case 2	Case 3	Case 4
Reference images	256 × 256 × 30	256 × 256 × 60	512 × 512 × 30	512 × 512 × 60
Moving images	256 × 256 × 30	256 × 256 × 60	512 × 512 × 30	512 × 512 × 60
